# Septic pulmonary embolism in China: clinical features and analysis of prognostic factors for mortality in 98 cases

**DOI:** 10.1186/s12879-019-4672-1

**Published:** 2019-12-27

**Authors:** Jing Jiang, Qiu-li Liang, Li-hua Liu, Shuang-qi Cai, Zhong-ye Du, Jin-liang Kong, Yi-qiang Chen

**Affiliations:** Department of Respiratory and Critical Care Medicine, The First Affiliated Hospital of Guangxi Medical University, Nanning, Guangxi 530021 People’s Republic of China No. 6 Shuangyong Road,

**Keywords:** Septic pulmonary embolism, Lung abscess, Bacteremia

## Abstract

**Background:**

To investigate the clinical features of septic pulmonary embolism (SPE) cases and prognostic factors for in-hospital mortality in China.

**Methods:**

A retrospective analysis was conducted of SPE patients hospitalized between January 2007 and June 2018 in the Department of Respiratory and Critical Care Medicine, The First Affiliated Hospital of Guangxi Medical University.

**Results:**

A total of 98 patients with SPE were identified. All patients had bilateral multiple peripheral nodules on chest computed tomography. The most common pathogen found in blood culture was *Staphylococcus aureus* (10/33, 30.3%). Transthoracic echocardiography was performed in 39 patients and 20 showed vegetations. Bronchoscopy was performed in 24 patients. Bronchoalveolar lavage fluid (BALF) was obtained from 15 patients (62.5%) and showed predominantly polymorphonuclear cell infiltration (52%, range of 48%~ 63%). Four patients received transbronchial lung biopsy, and histopathological examinations revealed suppurative pneumonia and organizing pneumonia. The in-hospital mortality rate was 19.4%. Age (odds ratio [OR] 1.100; 95% confidence interval [CI] 1.035–1.169), hypotension (OR 7.260; 95% CI 1.126–46.804) and ineffective or delay of empirical antimicrobial therapy (OR 7.341; 95% CI 1.145–47.045) were found to be independent risk factors for in-hospital mortality, whereas drainage treatment was found to be a protective factor (OR 0.33; 95% CI 0.002–0.677).

**Conclusions:**

SPE cases presented with nonspecific clinical manifestations and radiologic features. Blood cultures and bronchoscopy are important measures for early diagnosis and differential diagnosis. There is relationship between primary infection sites and the type of pathogen. Maintaining normal blood pressure and providing timely and appropriate initial antimicrobial therapy for effective control of the infection could improve prognosis.

## Background

Septic pulmonary embolism (SPE), unlike the much more common thrombotic pulmonary embolism, is a rare disorder in which a bacterial embolus containing microorganisms originating from the primary extrapulmonary infectious foci [1] (such as liver abscesses [2], peritonsillar abscesses [3], infection of skin and bone [4], infective endocarditis [5, 6], periodontal disease [7], intravascular devices and catheters [3]) obstructs the small pulmonary vasculature, causing sepsis and a focal abscess in the lung. SPE is associated with a high mortality rate and remains a diagnostic challenge in clinical practice due to its insidious onset, nonspecific clinical manifestations (such as fever, cough, dyspnea, chest pain and hemoptysis), and life-threatening complications [8]. Therefore, early diagnosis and appropriate treatment play important roles in reducing mortality [9]. However, histopathologic examination is rarely available in clinical practice, and there is a lack of unified diagnostic criteria and prognostic factors for SPE based on clinical, radiological and laboratory features [3]. The present study aimed to not only characterize the clinical features of SPE but also analyze the prognostic factors for mortality.

## Methods

### Data collection

Patient records from the Department of Respiratory and Critical Care Medicine at the First Affiliated Hospital of Guangxi Medical University from between January 1, 2007 to June 30, 2018 were retrospectively reviewed to identify cases of SPE. Hospitalized patients with SPE were identified using a computer-assisted search, and the following clinical data were extracted from their electronic medical records: age, sex, clinical course, symptoms at presentation, findings on physical examination, laboratory results, microbiologic culture results, and findings from chest radiography, echocardiography and bronchoscopy. SPE cases were identified according to the criteria reported by Cook et al. [3] with slight modification. The inclusion criteria were: (1) a chest computed tomography (CT) scan showing focal or multifocal lung infiltrates indicative of SPE; (2) the presence of active extra-pulmonary infection as potential embolic source; (3) exclusion of other potential explanations for the lung infiltrates; and (4) resolution of lung infiltrates with appropriate antimicrobial therapy or death due to septic shock. The exclusion criterion was age less than or equal to 14 years.

### Definitions

#### Hypotension

In this study, the following conditions were defined as hypotension: patients with systolic blood pressure (SBP) below 90 mmHg at admission, and excluded other physiological and primary hypotension.

#### Ineffective empirical antimicrobial therapy (EAT)

According to the guidelines of Community-Acquired Pneumonia (CAP) of Infectious Diseases Society of America/American Thoracic Society Consensus [10], ineffective ETA was defined as followed: After the initial EAT, (1) the condition of SPE was not improved, and the progress and deterioration require replacement of antimicrobial drugs, (2) or non-response was present despite EAT, suggesting the absence of or delay in achieving clinical stability (temperature < 37.8 °C, heart rate < 100 beats/min, respiratory rate < 24 beats/min, SBP ≥ 90 mmHg, PO2 ≥ 60 mmHg) [11, 12].

#### Statistical analysis

The data are expressed as mean ± standard deviation (SD) for continuous variables or median and interquartile range (IQR) for data with a skewed distribution, as well as frequencies and percentages for categorical variables. Continuous variables between the survival and mortality groups were compared using unpaired Student’s t test. Categorical variables were compared using the Pearson’s chi-squared test or Fisher’s exact test. Continuous variables among different infection sites were compared by variance analysis. To identify predictors of in-hospital mortality from demographic and clinical data, logistic regression was performed using clinically relevant variables that were found to differ significantly between the survival and mortality groups in the prior univariate analysis (*p* < 0.05). All statistical analyses were performed using SPSS software (v19.0, Chicago, IL, USA). *P* < 0.05 was considered statistically significant.

## Results

### Clinical characteristics

The study population consisted of 98 patients with a mean age of 46.63 ± 15.19 years (range 15–85 years), including 81 males and 17 females. Among those, 43 patients were current or ex-smokers (ever-smokers). The mean duration from the onset of symptoms to SPE diagnosis was 16.8 ± 6.9 days. The baseline clinical characteristics and univariate analysis of prognostic factors for in-hospital mortality of the 98 patients with SPE are shown in Table 1. The most common presenting symptom was fever (71.4%, including 40 patients with a high-grade fever defined as body temperature above 39 °C). Diabetes was the most common comorbidity. The most common predisposing condition was nosocomial infection, with skin and soft tissue infection (30.6%) being the most common foci of primary infection.

**Table 1:** Baseline characteristics, univariate analysis and Logistic regression analysis of prognostic factors for in-hospital mortality of the 98 patients with SPE

Characteristics/Variables	No. (%)/Value	Survival	Death	univariate analysis	Logistic regression analysis		
		(*n* = 79)	(*n* = 19)	P	P	OR	95% CI
Age (years)	46.6 ± 15.2	44.4 ± 14.0	56.0 ± 16.8	0.002	0.002	1.100	1.035–1.169
Sex (male/female)	81/17	66/13	15/4	0.635			
Smoking history	43 (43.9%)	34	9	0.733			
Presenting symptoms, n (%)							
2003Fever (> 38 °C)	70(71.4%)	56	14	0.808			
Cough	52(53.1%)	41	11	0.638			
Weight loss	27(27.6%)	19	8	0.114			
Dyspnea	23(23.5%)	16	7	0.126			
Chest pain	17(17.3%)	12	5	0.250			
Vital sign							
Hypotension (SBP < 90 mmHg)	13(13.3%)	6	7	0.001	0.037	7.260	1.126–46.804
Heart rate (> 100/min)	28(28.6%)	22	6	0.747			
Respiratory rate (> 22/min)	25(25.5%)	18	7	0.207			
Comorbidities							
Diabetes mellitus	41(41.8%)	33	8	0.979			
Heart disease	17(17.3%)	12	5	0.250			
Chronic liver disease (cirrhosis, hepatitis)	12(12.2%)	9	3	0.892			
Chronic kidney disease	11(11.2%)	7	4	0.268			
COPD	10(10.2%)	7	3	0.636			
Venous thromboembolism	9(9.2%)	8	1	0.828			
Deep vein thrombosis	6(6.1%)	6	0	0.593			
Pulmonary embolism	3(3.1%)	2	1	1.000			
Malignancy	5(5.1%)	4	1	1.000			
Collagen vascular disease or other autoimmune disorders	5(5.1%)	5	0	0.263			
Cerebrovascular disease	4(4.1%)	4	0	0.319			
Predisposing conditions							
Nosocomial infection	21(21.4%)	14	7	0.035	0.231	2.647	0.538–13.015
IV drug use	18(18.4%)	12	6	0.098			
Immunosuppressed state	16(16.3%)	12	4	0.783			
Venous catheter insertion	13(13.3%)	8	5	0.136			
Injury or other trauma	12(12.2%)	9	3	0.892			
Infectious foci							
Skin and other soft tissue infection	30(30.6%)	23	7	0.512			
Infective endocarditis	20(20.4%)	15	5	0.477			
Liver abscess	17(14.3%)	14	3	1.000			
Catheter-associated blood stream infection	9(9.2%)	6	3	0.504			
Urinary tract infection	6(6.1%)	5	1	1.000			
Perianal abscess	5(5.1%)	4	1	1.000			
Cholecystitis or cholangitis	3(3.1%)	3	0	1.000			
Infectious endophthalmitis	2(2.0%)	1	1	0.839			
Peritonsillar abscess	1(1.0%)	0	1	0.194			
Periodontal abscess	1(1.0%)	1	0	1.000			
Meningitis	1(1.0%)	0	1	0.194			
Unknown	3(3.1%)	3	0	1.000			
Laboratory data							
WBC count (×10^9^/L)	15.9 ± 7.3	15.9 ± 8.0	15.5 ± 2.9	0.818			
Neutrophil ratio	0.8 ± 0.1	0.8 ± 0.1	0.8 ± 0.2	0.873			
ESR, mm/hr	64.6 ± 30.2	63.7 ± 28.5	68.4 ± 36.8	0.547			
Serum CRP (mg/L)	84.0(55.9–135.0)	104.9 ± 63.1	89.5 ± 62.9	0.343			
Serum PCT (ng/ml)	1.36(0.29–6.52)	4.22 ± 2.18	5.74 ± 3.39	0.242			
PO_2_ (< 60 mmHg)	19(19.4%)	14	5	0.395			
Lactate level (> 2.0/mmol/L)	8(8.2%)	4	4	0.069			
Gram-positive organisms of blood culture	19(21.4%)	13	6	0.134			
Gram-negative organisms of blood culture	11(21.4%)	7	4	0.268			
Candidemia	3(21.4%)	0	3	< 0.001			
Deep fungal infection	14(14.3%)	8	6	0.016	0.233	0.193	0.013–2.885
MDRP infection	19(19.4%)	11	8	0.005	0.089	5.999	0.763–47.152
Clinical Course and complication							
Ineffective or delayed EAT	24(24.5%)	15	9	0.023	0.035	7.341	1.145–47.045
Drainage treatment	26(26.5%)	25	1	0.040	0.027	0.330	0.002–0.677
Respiratory failure	19(19.4%)	12	7	0.032	0.064	6.663	0.898–49.462
Heart failure	20(20.4%)	14	6	0.178			
Renal failure	14(14.3%)	9	5	0.095			
Liver insufficiency	10(10.2%)	7	3	0.636			
Coagulation disorders	4(4.1%)	1	3	0.026			
MODS	21(21.4%)	13	8	0.014	0.866	1.147	0.234–5.629
Duration of hospitalization, days	22.7 ± 11.1	21.4 ± 10.5	27.6 ± 13.8	0.037	0.895	1.005	0.935–1.080

Abbreviations: *SBP* Systolic blood pressure, *COPD* Chronic obstructive pulmonary disease, *IV* Intravenous, *WBC* white blood cell, *ESR* Erythrocyte sedimentation rate, *CRP* C reactive protein, *PCT* Procalcitonin, *PO*_*2*_: Partial pressure of oxygen, *EAT* Empirical antimicrobial therapy, *MODS* Multiple organ dysfunction syndrome

### Microbiologic findings

Both anaerobic and aerobic blood cultures were performed for all patients on the first day of hospitalization, and cultures of sputum, pus, urine, bronchoalveolar lavage fluid (BALF) and pleural effusion were also performed for some patients. A total of 85 pathogens were isolated from 58 patients, and in 17 of them, 2 or more pathogens were isolated from the same cases. The identified microorganisms are listed in Table 2. *Mycobacterium tuberculosis* was not isolated from any samples of the included patients. The positive blood culture rate was 33.7% (33/98). The most common pathogen found in the blood culture was methicillin-sensitive *Staphylococcus aureus* (MSSA, 6/33), followed by methicillin-resistant *Staphylococcus aureus* (MRSA, 4/33), *Viridans streptococci* (4/33) and *Klebsiella pneumonia* (4/33). In addition, different multidrug-resistant pathogens (MDRPs) were isolated from the blood, sputum, pus or BALF for 25 cases, including 7 with MRSA, 8 with extended spectrum β-lactamases (ESBL)-producing *Enterobacteriaceae*, 5 with multidrug-resistant *Pseudomonas aeruginosa* (MDR-PA), 4 with Carbapenem-resistant *Acinetobacter baumannii* (CR-AB), and 1 with vancomycin resistant *Enterococcus* (VRE).

**Table 2:** Microorganisms isolated from patients with SPE

Pathogens	Blood	Sputum	Pus	Urine	BALF	Pleural effusion	Total
	(*n* = 33)	(*n* = 24)	(*n* = 13)	(*n* = 6)	(*n* = 6)	(*n* = 3)	(*n* = 85)
Gram-positive (MDRP)	19(5)	4(1)	6(2)	0	0	1	30(8)
*Staphylococcus*	12	2	6			1	21
*Staphylococcus aureus* (MSSA/MRSA)	10 (6/4)	2 (1/1)	6 (4/2)			1	19 (12/7)
*Coagulase negative Staphylococcus*	1						1
*Staphylococcus epidermidis*	1						1
*Streptococcus*	6	2					8
*Viridans streptococci*	4						4
*Streptococcus haemolyticus*	2						2
*Streptococcus pneumoniae*		2					2
*Enterococcus faecalis* (VRE)	1(1)						1(1)
Gram-negative (MDRP)	11(5)	14(8)	7(2)	2	3(2)	1	38(17)
*Enterobacteriaceae*							
*Klebsiella pneumonia* (ESBL)	4(2)	5(2)	2		2(1)	1	14(5)
*Escherichia coli* (ESBL)	3(2)	1	3(1)	1			8(3)
*Enterobacter cloacae*	1	1		1			3
*Pseudomonas aeruginosa* (MDR-PA)	1(1)	3(2)	1(1)		1(1)		6(5)
*Burkholderia pseudomallei*	1		1				2
*Stenotrophomonas maltophilia*	1						1
*Acinetobacter A. baumannii* (CR-AB)		4(4)					4(4)
All MDRP	10	9	4	0	2	0	25
Fungus	3	6	0	4	3	1	17
*Candida*	3	4		4	1	1	13
*Saccharomyces albicans*		2					2
*Aspergillus*					2		2

*MDRP* Multidrug-resistant pathogen, *MRSA* Methicillin-resistant *Staphylococcus aureus, ESBL* Extended spectrum β-lactamases, *MDR-PA* Multidrug-resistant *Pseudomonas aeruginosa*, *CR-AB* Carbapenem-resistant *Acinetobacter baumannii*, *VRE* Vancomycin-resistant *Enterococcus*

### Radiological findings

**Fig. 1:**
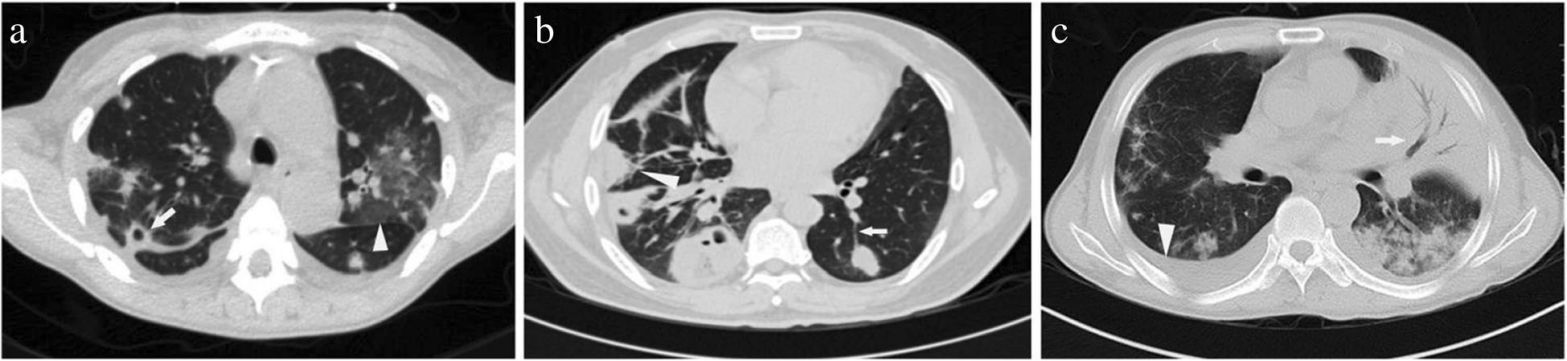
Chest CT revealed multiple peripheral nodules with cavitation (arrow, **a**) and ground-glass opacities (arrowhead, **a**); peripheral nodules with feeding vessel sign (arrow, **b**) and a wedge-shaped peripheral lesion (arrowhead, **b**), consolidation and air bronchogram (arrow, **c**) and pleural effusion (arrowhead, **c**)

Chest CT scans were available for all 98 patients. Representative images of the pathological findings in SPE cases are shown in Fig. 1. Multiple peripheral nodules were the most common radiographic finding and were seen in all 98 (100%) patients, followed by pleural effusion (64.3%), cavitation (59.2%), feeding vessel sign (28.6%), lobar consolidation (23.5%), peripheral wedge-shaped lesions (22.4%) and ground-glass opacities (18.4%). The diameter of the nodules ranged from 8 to 68 mm, and 83 patients (84.7%) had lesions with diameters between 10 and 30 mm.

### Echocardiography results

**Fig. 2:**
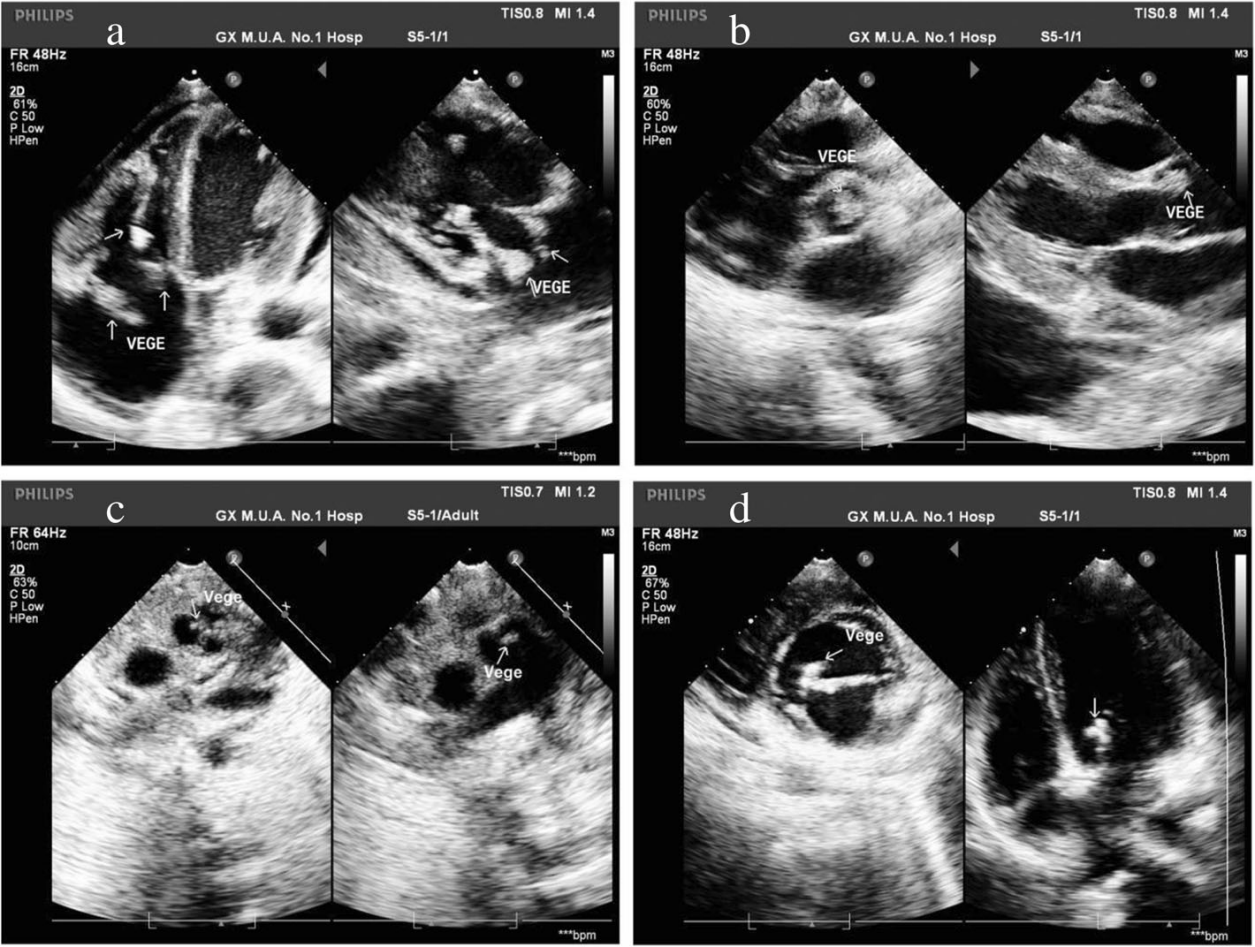
TTE demonstrated tricuspid valve and right ventricular vegetations (arrow, **a**); vegetations on the right coronary cusp of the aortic valve (arrow, **b**); vegetation on the pulmonary valve in a patient with congenital heart disease (arrow, **c**); and mitral valve vegetations (arrow, **d**)

Transthoracic echocardiography (TTE) was performed in 39 (39.8%) patients, and pathologies were identified in all cases. The identified pathologies were as following: (1) 20 patients had vegetations, including tricuspid valve vegetations in 8 patients (Fig. 2a), aortic valve vegetations in 5 patients (Fig. 2b), pulmonary valve vegetations in 2 patients (Fig. 2c), mitral valve vegetations in 2 patients (Fig. 2d), right atrium vegetations in 2 patients, and pacing lead in 1 patient; (2) mitral and aortic stenosis in 3 patients, with aortic, mitral and tricuspid regurgitations found in 19 patients; (3) right atrial thrombus in 2 patients; (4) congenital heart disease in 5 patients, including ventricular septal defect in 3 cases and patent ductus arteriosus in 2 cases; and (5) moderate and/or severe pulmonary hypertension in 14 patients.

**Fig. 3:**
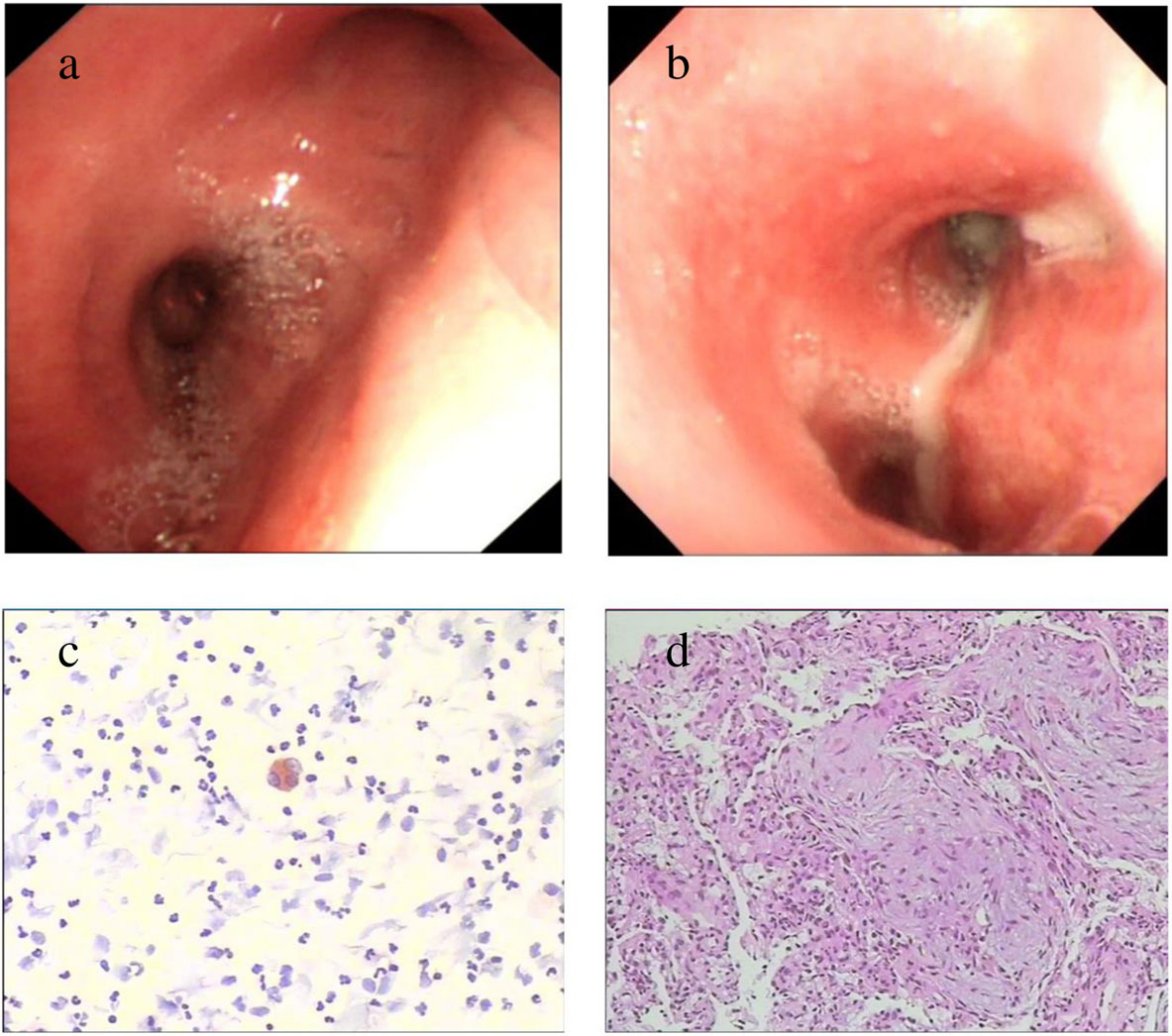
Bronchoscopy revealed hyperemia and edema of bronchial mucosa with serous secretion (**a**) and purulent secretion (**b**). The cytological examination showed massive PMN cell infiltration in BALF (**c**). Histopathology examination revealed organizing pneumonia (including widening of alveolar wall, infiltration of inflammatory cells in the lung interstitium, and disappearance of the alveolar cavity with replacement by hyperplastic fibrous tissue; **d**)

### Bronchoscopy examination outcomes

Twenty-four patients (24.5%) who did not present with the typical clinical picture of an infectious manifestation were examined by bronchoscopy. The most common findings were hyperemia and edema of the bronchial mucosa, which was seen in 20 patients (83.3%), followed by bronchial secretion in 11 patients (45.8%), including 8 cases of serous secretion (Fig. 3a) and 3 cases of purulent secretion (Fig. 3b). Bronchoalveolar lavage was performed in all 24 patients, and the differential cell counts in BALF showed predominantly polymorphonuclear (PMN) cells (52, 48%~ 63%) in 15 patients (62.5%), without signs of nuclear atypia or malignant cell (Fig. 3c). Moreover, four patients underwent transbronchial lung biopsy (TBLB), and the histopathological examinations revealed signs of suppurative pneumonia (purulent secretion filling the alveolar cavity and marked neutrophil infiltration) and organizing pneumonia (including widening of alveolar wall, infiltration of inflammatory cells in lung interstitium, and disappearance of the alveolar cavity with replacement by hyperplastic fibrous tissue), which are shown in Fig. 3d.

### Treatment outcomes and clinical courses

Most of the patients (65 patients, 66.3%) were treated with oral antibiotics before hospitalization. All patients received intravenous empirical antimicrobial therapy (EAT) at the hospital, including a combination of 2 compounds in 72 patients and a combination of 3 compounds in 26 patients. The most frequently prescribed antibiotics were cephalosporin (43.9%), followed by vancomycin (39.8%), carbapenem (meropenem and imipenem/cilastatin, 23.5%), piperacillin-tazobactam (20.4%), aminoglycosides (19.4%) and quinolones (12.2%). Moreover, 26 (26.5%) patients received antifungal therapy, both based on the positive microbiologic findings (16 patients) and prophylactically (10 patients). The antifungal agents administered were fluconazole (17 patients, 17.3%), voriconazole (6 patients, 6.1%) and echinocandin (3 patients, 3.1%). The total duration of antibiotic therapy including oral antibiotic use after discharge was 48.3 ± 22.6 days. Twenty-four patients (24.5%) received either ineffective EAT (20 patients) and had to change antimicrobial therapy after blood culture results were obtained, or appropriate antimicrobial treatment was delayed for 48 h (4 patients) after their blood culture results were obtained.

Moreover, 28 (28.6%) patients underwent thoracentesis, and 26 patients required pigtail drainage of a liver abscess (14 patients, 14.3%), skin abscess (7 patients, 7.1%), empyema (3 patients, 3.6%) or perianal abscess (2 patients, 2.0%). Six (6.1%) patients with IE underwent cardiac surgery, including ventricular septal defect repair surgery in 3 cases and tricuspid valve replacement surgery in 3 cases. Among those 6 cases, 4 patients survived. Of the other 14 patients with IE who did not undergo cardiac surgery, 3 died during hospitalization and 11 were discharged from hospital. Among those discharged, 3 of them died of severe heart failure and recurrence of infection within six months after discharge. Eight (8.2%) patients received systemic anticoagulants due to thromboembolic disease.

Finally, SPE was cured in 18 patients (18.4%), while 55 cases (56.1%) showed improved and 25 cases (25.5%) required admission to the intensive care unit (ICU) due to the patients’ critical condition and requirement of ventilator support. Among those admitted to the ICU, 19 patients died due to fatal complications, including shock (6 patients), respiratory failure (7 patients), heart failure (6 patients), renal failure (5 patients), liver insufficiency (3 patients), coagulation disorders (3 patients), multiple organ dysfunction syndrome (MODS, 8 patients) and neurological complications (1 patient). The median duration of hospitalization was 23.5 days (range 12.0–34.8 days).

### Clinical characteristics by each primary infection of the 98 patients with SPE

The baseline characteristics grouped according to the different primary infections of the 98 patients with SPE was shown in Table 3. The type of pathogens differ dependent on the different types of primary infection and the relationship was found to be statistically significant (*P* < 0.05). Gram-positive bacteria were more frequently found in SPE patients with skin and soft tissue infection and infective endocarditis, while Gram-negative bacteria were more frequently found in SPE patients with liver abscess. However, there was no difference in the frequency of MDRP were found among the different primary infections of SPE patients (*P* > 0.05). Moreover, no difference in age, diabetes or the duration of hospitalization was found among the different primary infections of SPE patients (*P* > 0.05).

**Table 3:** Baseline characteristics by each primary infection of the 98 patients with SPE

Primary infection foci	Skin and other soft tissue infection (*n* = 30)	Infective endocarditis (*n* = 20)	Liver abscess (*n* = 17)	Catheter-associated blood stream infection (*n* = 9)	Urinary tract infection (*n* = 6)	Perianal abscess (*n* = 5)	Other infection And unknown (*n* = 11)
Age, years^§^	49.1 ± 17.0	38.8 ± 14.2	46.8 ± 13.3	52.2 ± 14.3	51.0 ± 8.0	52.5 ± 10.4	46.2 ± 18.0
Pathogens*							
Gram-positive	16	8	2	2	1	0	1
Gram-negative	14	2	12	3	4	2	1
Fungus	6	2	1	3	5	0	0
MDRP**	11	2	6	4	0	2	0
Predisposing conditions							
Diabetes mellitus**	14	8	9	3	3	1	3
Nosocomial infection	7	3	3	4	2	1	1
IV drug use	4	6	3	2	1	2	0
Immunosuppressed state	5	3	3	2	2		1
Injury or other trauma	4	2	2	1	1	1	1
Antimicrobial therapy							
Cephalosporin	17	10	7	2	1	2	4
Vancomycin	19	11	0	6	1	0	2
Carbapenem	6	5	4	4	1	1	2
Piperacillin-tazobactam	4	4	6	1	2	1	2
Aminoglycosides	0	0	10	1	4	4	0
Quinolones	2	2	2	0	4	0	2
Antifungal therapy	8	4	2	4	5	0	3
Drainage	7		14	1	2	2	0
Death	6	4	3	3	1	1	1
Duration of hospitalization, days^§^	23.4 ± 12.1	19.9 ± 9.4	22.3 ± 9.4	24.1 ± 16.2	17.3 ± 6.4	32.1 ± 11.0	20.8 ± 6.0

^§^There are no significant relationship between age as well as duration of hospitalization and different primary infection of SPE patients, *P* > 0.05

* There is relationship between pathogens and different primary infection sites of the SPE patients, *P* < 0.05

** There is no relationship between MDRP as well as diabetes and the different primary infection of SPE patients, *P* > 0.05

*MDRP* Multidrug-resistant pathogen

### Prognostic factors for in-hospital mortality among patients of SPE

The in-hospital mortality rate for SPE patients in the present study was 19.4% (19 patients). To identify the prognostic factors for in-hospital mortality in patients with SPE, a univariate analysis of clinical and laboratory parameters was conducted. The results identified the following factors as significantly associated with in-hospital mortality: age, duration of hospitalization, hypotension, nosocomial infection, candidemia, deep fungal infection, MDRP infection, ineffective or delayed EAT, drainage treatment, respiratory failure, coagulation disorders and MODS (*P* < 0.05, Table 1). Entry of these factors into a logistic regression analysis revealed that the independent risk factors for mortality were age (odds ratio [OR] 1.100; 95% confidence interval [CI] 1.035–1.169), hypotension (OR 7.260; 95% CI 1.126–46.804) and ineffective or delayed EAT (OR 7.341; 95% CI 1.145–47.045), whereas drainage treatment was a protective factor against mortality (OR 0.33; 95% CI 0.002–0.677, Table 1).

## Discussion

The present study retrospectively analyzed a large cohort of 98 SPE patients. The results showed that fever was the most common clinical symptom and MSSA was the most common pathogen in blood culture. Moreover, bilateral multiple peripheral nodules were seen on chest CT scans, and echocardiography was performed in some of the patients, suggesting vegetations. These results agree well with previous studies reported in the literature [3, 4, 6, 13]. However, the clinical characteristics of the patients included in the present study differed from those of patients in previous reports, including the most common infectious foci and comorbidities. Furthermore, bronchoscopy examination of some SPE patients demonstrated acute bronchitis, suppurative pneumonia and organizing pneumonia, which were not reported previously. Finally, the present study found that the independent risk factors for SPE mortality were age, hypotension and ineffective or delayed EAT, whereas drainage treatment was a protective factor against mortality. In contrast to a previous study, tachypnea (respiratory rate > 22/min) was not found to be an independent risk factor for mortality [14].

Historically, SPE was known as a deadly complication in the pre-antibiotic era following septic pelvic thrombophlebitis due to either septic abortion or postpartum uterine infection [15]. In the 1970s, the epidemiology of the disease was changed with the use of catheters, and it was discovered that many patients suffering from SPE had infective endocarditis due to intravenous drug abuse [8] [15]. In the 2000s, Cook et al. reported that Lemierre syndrome and central venous catheter infection were the common causes of SPE [3]. Moreover, two studies from Taiwan and one from Japan reported on SPE cases caused by liver abscesses [16, 17] and periodontal disease [7], respectively. Recently, several studies demonstrated that bone, skin, and soft tissue infections are the most common source of infection [4, 6, 14]. Similarly, the present study also found that skin and soft tissue infection (30.6%) was the most common foci of primary infection, followed by infective endocarditis (IE, 20.4%). Unfortunately, the present study did not study the management of these primary infections in detail since diagnosis and management of the primary infections was carried out by consultants from corresponding departments.

In the present study, almost half of the patients had a diabetes diagnosis (41.8%), which was much higher than the previously reported incidences of 16.7, 17.1 and 28.6% [4, 6, 7]. This could be related to the increased incidence of diabetes in China in the recent years. Interestingly, we found that the incidence of diabetes was as high as 82.4% in patients with SPE caused by liver abscess, similar to two other studies from Taiwan, which reported incidences of 66.7 and 85.7% [2, 17]. However, no studies have reported diabetes as an independent risk factor for mortality in SPE, probably because most patients with diabetes can receive timely and effective treatment to keep their blood glucose under control after diagnosis.

Echocardiography could aid the diagnosis of IE which can eventually lead to SPE. A total of 39 (39.8%) patients received echocardiography in this study, which was lower rate than other similar studies (57.1–100%) [3, 4, 6, 13, 14]. The reasons for this were as follows: 1. Some patients did not undergo echocardiography because SPE and their primary infection foci were clearly diagnosed and there was no reason to suspect IE and comorbidity of heart disease; 2. Some patients were initially misdiagnosed as other diseases without undergoing echocardiography, but later improved on antibiotic treatment against IE and because of the lack of basic heart disease, echocardiography was not performed; 3. Some patients refused to undergo echocardiography for financial reasons (some medical insurance does not cover this examination) despite recommendation by doctors.

The present study was, to our best knowledge, the first to describe bronchoscopy findings of SPE patients in detail. The purpose of bronchoscopy and TBLB was to determine the pathogen in infectious pulmonary diseases and to exclude other pulmonary diseases. However, it would be unethical to perform this procedure in every single patient not every patient because it is a high-risk procedure for SPE patients, with risks such as fever, dyspnea, hemoptysis and pneumothorax, and the procedure is therefore only reserved for the following patients: (1) who did not reveal culture results from blood and primary foci, (2) who did not present with typical infectious manifestation, (3) to exclude other pathology such as pulmonary TB and cancer with lung to lung metastasis, (4) who did not have fever, pulmonary hypertension, coagulopathy, organ dysfunction and other contraindications, (5) TBLB was not performed on lesions through chest CT scan, including cavitary septic foci or abscess, that could lead to pneumothorax [18]. In our study, only 24 patients with atypical manifestation underwent bronchoscopy and 4 patients underwent TBLB. The results from differential cell counts and microbial culture of BALF as well as lung biopsy were consistent with deep airway infectious disease. Besides, CT-guided or ultrasound-guided percutaneous needle biopsy have also been reported to be effective diagnostic methods for peripheral pulmonary lesions (PPLs), with a high diagnostic rate of 81.8–97% and acceptable complication rate of 8–17% [19, 20]. In this study, 4 patients with PPLs refused the above two examinations because of the results of enhanced CT and ultrasound, which showed abundant blood vessels and high risk of hemoptysis. Furthermore, endobronchial ultrasound (EBUS)-guided transbronchial biopsy (TBB) has a high diagnostic accuracy of 61.8–77% [21, 22]. Unfortunately, our hospital only had short experience with this procedure and lacked sufficient clinical data. Increased use of interventional examinations could improve the diagnosis rate of SPE patients without typical infection manifestation in the future.

Some studies have suggested that septic shock and heart failure are both independent risk factors of IE [23, 24]. However, among the patients who had both IE and heart disease in the present study, the hypotension seen in some patients could not be explained by either infection or heart failure and could lead to septic shock and heart failure. Therefore, hypotension was evaluated as a risk factor for SPE mortality. Consistent with the previous results, the present study showed a significant increase in the risk of mortality if patients remained hypotensive after appropriate antibiotics and therapy for heart failure.

Antimicrobial therapy plays an important role in SPE treatment. It is well known that inappropriate initial antibiotic treatment, including delayed administration of antibiotics and ineffective EAT, increases mortality in sepsis or septic shock cases [25–27]. Similarly, our analysis showed that SPE patients who received ineffective or delayed EAT had a seven-fold increased risk of mortality. Although there is a lack of standardized SPE guidelines, we believe that appropriate antibiotic treatment should be given as soon as possible, similar to the recommendation for sepsis patients with or without septic shock [28–30], because SPE is essentially a pulmonary infection caused by bacteremia resulting from another primary infectious site. Although our findings showed that the most common pathogen was *MSSA*, the positive rates of Gram-negative bacteria (*Klebsiella pneumonia* and *Escherichia coli*) as well as fungal organisms were also high. These results suggest that both Gram-positive and Gram-negative bacteria should be covered with broad-spectrum antibiotics when choosing the initial EAT and antifungal therapy should also be administered. The choice of EAT can be based on a patient’s history, the anatomic site of infection, the patient’s immunosuppression status and local microbiology prevalence [31–34] and adjusted according to results of microbial culture if EAT is ineffective. However, there are no clear guidelines on the optimal duration of the course of antibiotic therapy for SPE patients. In the present study, the mean duration of total antibiotic therapy was 48.3 ± 22.6 days, which was consistent with the 4–8 weeks reported in previous publications [3, 4, 6, 13]. The decision to stop antibiotics can be based on clinical improvement, subsequent culture results, levels of inflammatory indicators and radiological findings [35].

Clinical experience suggests that it is difficult to achieve control and improve the symptoms of systemic infection by administering antibiotics if patients do not receive timely and effective control of the primary infection locally, such as the drainage of an abscess, debridement of infected necrotic tissue, and removal of a potentially infected device [16, 36–38]. Some studies have suggested that drainage therapy for infectious foci should be given together with antibiotics, but no further analysis of the effect of drainage on SPE mortality has been reported [2, 3, 6, 17]. The present study has now confirmed that drainage of the infectious foci is a protective factor against mortality in SPE. Patients who received drainage had a lower risk of death. Therefore, we recommend drainage of abscesses as soon as possible once a specific primary infection is identified.

Some studies suggested that cardiac surgery is an independent protective factor that improves the prognosis in IE patients [23, 24, 39]. However, due to the small sample size in the present study, we were unable to assess the relationship between cardiac surgery and SPE mortality. Among the six patients who received cardiac surgery in the present study, the survival rate was relatively high (66.7%). However, a prospective study with a larger sample size is required to more closely assess this relationship.

In addition, we speculated that deep fungal infection (particularly candidemia), infections caused by MDRPs and severe coagulation disorders could also be risk factors for SPE mortality, because these conditions are more difficult to control clinically. The results of univariate analysis of candidemia and coagulation disorders between the mortality and survival groups were consistent with this hypothesis. However, these two factors were not included in the logistic regression analysis due to the small sample size. Deep fungal infection and infection caused by MDRPs were on the other hand included in the logistic regression analysis, because they were shown to differ significantly between the mortality and survival groups in the univariate analysis. However, they were not identified as independent risk factors. Nevertheless, we recommend that SPE patients with risk factors for invasive fungal infection, such as immunosuppressed status, long-term use of broad-spectrum antibiotics, prolonged hospital/ICU stay, prolonged invasive vascular catheters, receipt of parenteral nutrition and use of H2 blockers should receive treatment with antifungal drugs empirically to avoid a poor prognosis [40, 41]. At the same time, attention should be paid to the rational use of antibiotics to reduce the incidence of MDRP infections.

MDRP is a growing issue globally that endangers the lives of many patients. Twenty-five cases of MDRP were found in the present study. No relationship between MDRP and the type of primary infection was found. The occurrence of MDRP is increased in the presence of the following risk factors: prolonged hospital stay, recent use of antimicrobial agents, prior hospitalization, and prior colonization or infection with MDRP [35]. These risk factors should be considered in the choice of empirical antibiotic therapy. For examples, if there are risk factors for MDRP in patients with skin and soft tissue infection and IE, in which the Gram-positive bacteria is most commonly the pathogenic agent, MRSA infection should be considered and empirical anti-MRSA agent, such as vancomycin, linezolid or teicoplanin, could be used when the condition is critical.

The present study has several limitations. First, this study was a single-center retrospective study, which inevitably results in bias. Second, the positive rate of blood cultures was much lower than those reported in developed countries (70–90%) [3, 4]. This was most likely because many of the patients had already received antibiotics before admission to the hospital where blood culture was performed. It was therefore difficult to gather enough data to conduct a good statistical analysis. Third, TTE was only performed in a limited number of SPE patients, and none underwent transesophageal echocardiography, which could have led to some missed IE cases. Finally, the present study lacked follow-up data. Only in-hospital mortality was analyzed as it was not possible perform a survival analysis and explore the impact of prognostic factors on overall mortality.

## Conclusions

In conclusion, the present study demonstrated that SPE should be suspected in high-risk patients who present with typical clinical manifestations (fever, dyspnea, chest pain) and radiologic features (particularly multiple nodules in bilateral lobes on chest CT). Undergoing blood culture before the use of antibiotics and identifying primary infection foci are important to make early diagnosis of SPE. Gram-positive bacteria are responsible for a higher proportion in SPE patients with skin and soft tissue infection and infective endocarditis, while Gram-negative bacteria are mainly found in SPE patients with liver abscess. Bronchoscope is strongly recommended to exclude noninfectious diseases, if patients present with atypical infectious symptoms. Extra attention should be given to SPE patients over 56 years old, those who are hypotensive and those who received ineffective or delayed EAT, as they could be at high risk for in-hospital mortality. Timely drainage treatment of infectious foci could reduce mortality.

## Data Availability

The datasets generated and analyzed during the present study are available from the corresponding author on reasonable request.

## Abbreviations

BALF: Bronchoalveolar lavage fluid
CI: Confidence interval
COPD: Chronic obstructive pulmonary disease
CR-AB: Carbapenem-resistant *Acinetobacter baumannii*
CRP: C reactive protein
CT: Computed tomography
EAT`: Empirical antimicrobial therapy
ESBL: Extended spectrum β-lactamases
ESR: Erythrocyte sedimentation rate
ICU: Intensive care unit
IE: Infective endocarditis
IQR: Interquartile range
IV: Intravenous
MDR-PA: Multidrug-resistant *Pseudomonas aeruginosa*
MDRPs: Multidrug-resistant pathogens
MODS: Multiple organ dysfunction syndrome
MRSA: Methicillin-resistant *Staphylococcus aureus*
MSSA: Methicillin-sensitive *Staphylococcus aureus*
OR: Odds ratio
PCT: Procalcitonin
PMN: Polymorphonuclear
PO_2_: Partial pressure of oxygen
SBP: Systolic blood pressure.
SD: Standard deviation
SPE: Septic pulmonary embolism;
TTE: Transthoracic echocardiography
VRE: Vancomycin resistant *Enterococcus*
WBC: White blood cell
